# Antiretroviral effect of lovastatin on HIV-1-infected individuals without highly active antiretroviral therapy (The LIVE study): a phase-II randomized clinical trial

**DOI:** 10.1186/1745-6215-10-41

**Published:** 2009-06-18

**Authors:** Carlos J Montoya, Fabian Jaimes, Edwin A Higuita, Sandra Convers-Páez, Santiago Estrada, Francisco Gutierrez, Pedro Amariles, Newar Giraldo, Cristina Peñaloza, Maria T Rugeles

**Affiliations:** 1 Immunovirology Group, University of Antioquia, Medellin, Colombia; 2 Department of Internal Medicine and Grupo Academico de Epidemiologia Clinica, School of Medicine, University of Antioquia, Medellin, Colombia; 3 Grupo Academico de Epidemiologia Clinica, School of Medicine, University of Antioquia, Medellin, Colombia; 4 Laboratorio Clinico Congregacion Mariana, Medellin, Colombia; 5 Faculty of Pharmaceutical Chemist, University of Antioquia, Medellin, Colombia; 6 Research Group on Pharmaceutical Prevention and Promotion, Department of Pharmacy, University of Antioquia, Medellin, Colombia; 7 Humax Pharmaceutical, Medellin, Colombia; 8 Laproff Laboratories, Medellin, Colombia

## Abstract

**Background:**

Highly active antiretroviral therapy produces a significant decrease in HIV-1 replication and allows an increase in the CD4 T-cell count, leading to a decrease in the incidence of opportunistic infections and mortality. However, the cost, side effects and complexity of antiretroviral regimens have underscored the immediate need for additional therapeutic approaches. Statins exert pleiotropic effects through a variety of mechanisms, among which there are several immunoregulatory effects, related and unrelated to their cholesterol-lowering activity that can be useful to control HIV-1 infection.

**Methods/design:**

Randomized, double-blinded, placebo controlled, single-center, phase-II clinical trial. One hundred and ten chronically HIV-1-infected patients, older than 18 years and naïve for antirretroviral therapy (i.e., without prior or current management with antiretroviral drugs) will be enrolled at the outpatient services from the most important centres for health insurance care in Medellin-Colombia. The interventions will be lovastatin (40 mg/day, orally, for 12 months; 55 patients) or placebo (55 patients). Our primary aim will be to determine the effect of lovastatin on viral replication. The secondary aim will be to determine the effect of lovastatin on CD4+ T-cell count in peripheral blood. As tertiary aims we will explore differences in CD8+ T-cell count, expression of activation markers (CD38 and HLA-DR) on CD4 and CD8 T cells, cholesterol metabolism, LFA-1/ICAM-1 function, Rho GTPases function and clinical evolution between treated and not treated HIV-1-infected individuals.

**Discussion:**

Preliminary descriptive studies have suggested that statins (lovastatin) may have anti HIV-1 activity and that their administration is safe, with the potential effect of controlling HIV-1 replication in chronically infected individuals who had not received antiretroviral medications. Considering that there is limited clinical data available on this topic, all these findings warrant further evaluation to determine if long-term administration of statins may benefit the virological and immunological evolution in HIV-1-infected individuals before the use of antiretroviral therapy is required.

**Trial registration:**

Registration number NCT00721305.

## Background

Type-1 human immunodeficiency virus (HIV-1), the etiologic agent of the acquired immunodeficiency syndrome (AIDS), causes a chronic disease characterized by a progressive loss of CD4+ T cells associated with other quantitative and qualitative alterations of the immune response. Currently, HIV-1 infection is among the most important public health problems around the world: at the end of 2007, there were around 33 million people infected, 2,5 million new cases were diagnosed and 2,1 million HIV-1-infected individuals died as a consequence of HIV-1-derived clinical complications [1]. In Colombia, is has been estimated that more than 190.000 persons are infected with HIV-1 (calculated prevalence: around 0.7%), and 24.000 deaths due to HIV-1 infection were reported between 1987 and 2007 [1,2].

The dysregulation of the immune response appears early during HIV-1 infection when individuals gradually lose their T lymphocyte proliferative responses to recall antigens, alloantigens and mitogens, before the severe reduction in CD4 T-cell count appears. In addition to the direct elimination of CD4 T cells, HIV-1 impair immune function directly through the immunosuppressive effect of viral proteins and by a state of uncontrolled immune activation that leads to immunosuppression and accelerated CD4 T-cell death [3-5].

The current treatment for HIV-1 infection is highly active antiretroviral therapy (HAART), which decreases viral replication and plasma HIV-1 RNA levels, allowing partial immune restoration that associates with a decrease in the incidence of opportunistic infections and mortality [6]. However, after HAART is discontinued there is a rebound in viral load and a decrease in CD4+ counts to similar levels than before HAART administration. In addition, potent antiretroviral therapy administered for several months is unable to eliminate HIV-1 tissue reservoirs effectively and do not lead to a full recovery of the immune response [7].

On the other hand, international guidelines for HAART therapy in chronically HIV-1-infected adults recommend beginning these regimens in those individuals with an AIDS-defining illness, in HIV-1 positive pregnant women, and in patients with a CD4+ T-cell count lower than 350 cells/μL [8]. In this sense, too many patients with an early diagnosis of HIV-1 infection would be without antiretroviral therapy during several years, with a persistent viral replication and a progressive lymphoid tissue damage, which may affect the level of immune restoration achieved after the late onset of HAART. Furthermore, the cost and complexity of HAART regimens, the recommended delayed beginning of HAART in adults chronically infected with HIV-1, the growing list of long-term side effects, and the eventual development of resistance have underscored the immediate need for additional therapeutic approaches.

Statins are mainly known for their plasma cholesterol-lowering properties and are widely used for the prevention of cardiovascular diseases [9,10]. They, however, also have pleiotropic actions through a variety of mechanisms, among which there are several virological and immunoregulatory effects that are unrelated to their cholesterol-lowering activity [11-13]. Depletion of cholesterol alters the capacity of a cell to form lipid rafts, and numerous studies have shown that HIV-1 requires lipid rafts for several key stages of its replication cycle [14,15]. Moreover, some evidence suggests that statins alter the intracellular signals dependent on Rho GTPases [16], the functional activity of LFA-1/ICAM-1 adhesion molecules [17], the expression of the CCR5 coreceptor and the secretion of RANTES [18], and may exert significant modulatory effects on the balance of the cytokine network in human beings [19,20]. Based on these immunomodulatory properties, statins are being evaluated in the treatment of diseases beyond classic cardiovascular conditions [21]. Therefore, statins therapy might modulate the immune system function in HIV-1-infected individuals, affecting the clinical course of this infection. Whether these observations translate into significant clinical interactions is uncertain, and very limited clinical data are available on this topic.

We hypothesize that the long-term use of statins will benefit the overall evolution of HAART naïve HIV-1-infected individuals, having the following effects: i) decreasing plasma viral load; ii) increasing blood CD4 T-cell count; iii) restoring the immune system function; and iv) decreasing the frequency of morbidity and mortality. To evaluate this hypothesis, this investigation is aimed to explore the effect of lovastatin (oral intake, 40 mg/day during one year) on viral load and CD4 T-cell count, adaptive immune parameters, cholesterol metabolism and Rho GTPases function, and clinical evolution of chronically HIV-1-infected individuals who are HAART naïve.

## Methods/design

### Study Design

Randomized, double-blinded, placebo-controlled, phase II clinical trial.

### Study Population

One hundred and ten (110) chronically HIV-1 infected patients without antiretroviral treatment.

The inclusion criteria will be the following:

- Asymptomatic HIV-1 positive individuals, with age ≥ 18 years, who are HAART naive

- HIV-1 infection confirmed by: a) any positive western blot at least six months before admission to the study; or b) within the last six months by a western blot that includes the p31 and p66 bands

- Detectable viral load but less than 100,000 copies/mL

- CD4+ T cell count ≥ 350 cells/μL

The exclusion criteria will be the following:

- Inability or unwillingness of patients to give written informed consent

- Main residence outside Medellin and its metropolitan area, or any indication of difficulties in the follow-up period

- Participation in other clinical trials

- Evidence that the patient will exhibit low adherence to intervention and follow-up (Morisky-Green test)

- Pregnancy or breastfeeding

- Any type of antiretroviral treatment before admission to the study, and therapy with lipid-lowering drugs during the last six months

- Antecedents of allergy, contraindications or intolerance to statins

- Patients receiving medications which can generate relevant interactions with lovastatin: clarithromycin, erythromycin, azithromycin, itraconazole, ketoconazole, nefadozone, cimetidine, rifampin, phenobarbital, carbamacepin, phenitoin, gemfibrozil.

- Unwillingness to avoid the consumption of Citrus paradise (grapefruit juice) or Saint John's Wort (Hypericum)

- Chronic active hepatitis (B or C)

- Any hepatocellular disease, indicated by elevation of liver enzymes (AST or ALT) more than twice the upper limit of reference values

- Renal failure, indicated by serum creatinine ≥ 2 mg/dL

- Myopathy, indicated by an elevation of creatine phosphokinase (CPK) more than five times the upper limit of reference values

- Infection or acute disease that requires inpatient treatment

- Active substance-related disorders.

- Medical indication for using lipid-lowering drugs

### Randomization

The treatment assignment ratio will be 1:1, fixed throughout the study. The allocation sequence will be developed by a statistician in the Data Coordinating Center (DCC) using randomly permuted blocks of size 2, 4 and 6 generated by a random number generator (*ralloc *program, Stata co. 8.2, College Station, TX, USA). Once the sequence is generated, it will be matched with sequential numbers between 001 and 110 and given to the manufacturer for labelling the blisters containing the pills with lovastatin or placebo. The allocation sequence will remain in a confidential file in the DCC unknown for the clinician investigators, the patients, and the pharmacists who dispensed the interventions. This sequence will be disclosed only after study termination for data analysis, or at request by the Data Safety Monitoring Board (DSMB).

### Allocation concealment mechanism

The manufacturer will deliver both interventions (lovastatin and placebo) to the clinical investigators labelled only with the number code assigned from the list given by the DCC. Neither the investigators nor the pharmacists will know the actual content of the pills, as they will be contained in identical blisters and their physical characteristics will be the same (size, shape, colour and smell). The complete treatment course for one year, dispensed monthly, will be packed in sealed boxes equally labelled with the corresponding number, unique for each participant. Each treatment will be allocated and opened only for the sequential participant intended to enrol after the verification of eligibility criteria. The pharmacists will handle the interventions and will be responsible for their maintenance under lock and key. The dosage for lovastatin is 40 mg per day, represented by two pills to take each night; for this reason both interventions will be dispensed to fulfil two pills per night.

### Clinical follow-up

Before the enrolment and monthly during the study, all the patients will have a complete clinical evaluation performed by a physician who is blinded for the intervention assigned to each patient. Before the intervention (day 0) and 30, 180 and 360 days of study, blood samples will be collected to perform laboratory analysis (transaminases, CPK, serum lipid profile). The intervention will be stopped in presence of serious adverse events, a three-fold increase in serum transaminases or a five-fold increase in CPK. In order to preserve the blinding process, only the first value of serum lipid profile (day 0) will be known by the clinical investigators (eligibility criteria). Afterwards, for the samples at 180 and 360 days of following, a medical safety monitor in the DCC will receive the report directly from the laboratory.

The following clinical and pharmacotherapy parameters will be evaluated monthly for one year, during the medical control visits:

- Tolerance to medications and adverse events

- Adherence to therapy (lovastatin or placebo) and prevention of withdrawals

- Incidence of infections (opportunistic and non-opportunistic)

- Incidence of non-infectious diseases defining AIDS

- Hospitalizations due to diseases related to progressive HIV-1-infection

- Death due to diseases related to progressive HIV-1-infection

### Interventions and safety of statins

After the first medical appointment and then monthly, each participant will receive the whole therapy for 1 month (60 tablets of lovastatin or placebo) from the pharmacist who also evaluates adherence to the intervention and gives usage recommendations. The statins are well tolerated by most persons; they have proven to be extremely safe in the vast majority of patients. Few significant side effects have been observed in clinical trials, and post-marketing reports of adverse events have been very limited when considered in comparison to a very large number of persons safely using them. The main risks of statins are elevation of hepatic enzymes, and myopathy. Elevated hepatic transaminases generally occur in 0.5% to 2.0% of patients and are a dose-dependent effect; progression to liver failure specifically due to statins is exceedingly rare. Myopathy induced by stain is also rare. Mild gastrointestinal symptoms (e.g., constipation, flatulence, dispepsia, and abdominal pain), are the most common adverse effects of lovastain. Thus, a lovastatin dose of 40 mg/day is well tolerated with low discontinuance rates (lower than 2%). [22]

### Retention and adherence to the trial

The study is conducted in the outpatient services from the most important centres for health insurance care in the city of Medellín (northwest region of Colombia). The staff physicians are informed of the study protocol and they are attentive to eligibility criteria in their daily consultation. Once a potential participant is detected, her/his attending physician will give her/him information regarding the clinical trial and ask for permission to give both complete identification (full name, identity number) and contact phones (main and alternative) to the trial coordinators. They will call those potential participants and invite them to an informative meeting with the clinical investigators. In this interview, eligibility criteria should be applied including laboratory screening. When the results come out, they are sent to the trial coordinator who should again contact the potential participant for a recruiting visit, in which informed consent will be signed.

To keep permanent contact with potential and recruited participants, the trial coordinator will be available through a 24-hour mobile phone and e-mail messaging. Also, she should maintain a constant two-way communication with the HIV-attention program to constantly detect new potential candidates. At the end of each consultation, the next appointment will be scheduled in advance, giving a card with emergency phones and information. All participants will be contacted by the trial coordinator at least two days before the scheduled visit to confirm it and to re-schedule, if necessary. If any of the potential or recruited participants are not available, an active search for updated contact information should be performed beginning by reviewing the health insurance database and also calling the alternative phone numbers provided. Special attention should be given to the basal condition of individuals at the time of the programmed laboratory tests. Recommendations are given in each consultation, and the participants will be encouraged to call the trial coordinator to solve doubts or questions any time.

### Study outcomes

The efficacy of lovastatin to control HIV-1 replication allowing an improved immune reconstitution will be determined by primary and secondary outcomes. The primary outcome will be the viral load measured as RNA copies per mL of peripheral blood, and the secondary outcome will be the CD4 T-cell count measured as cells per μL of peripheral blood; both determined at enrolment, and 6 and 12 months after the intervention.

The tertiary or exploratory outcomes will be the following (determined at enrolment, and 6 and 12 months of interventions, unless indicated otherwise):

- Absolute number per μL of peripheral blood of CD8+ T lymphocytes and the CD4/CD8 ratio

- Basal expression of immunological activation markers (CD38 and HLA-DR) on CD4+ and CD8+ T lymphocytes

- Concentration of total serum cholesterol

- Concentration of cellular cholesterol

- Functional activity of LFA-1 and ICAM-1

- Activity of Rho GTPases

- Monthly frequency and type of: infections, non-infectious AIDS defining diseases, hospitalizations

- Mortality due to an HIV-1/AIDS related cause

### Laboratory assays

#### Viral load

The plasma HIV-1 viral load will be determined using the commercial assay "RT-PCR Cobas Ampliprep-Cobas Amplicor" (Roche, Indianapolis, IN).

#### Flow cytometry

Mouse fluorochrome-labeled monoclonal antibodies (mAbs) against the following human molecules will be used: CD3, CD4, CD8, CD38 and HLA-DR; these antibodies and the corresponding isotype control antibodies will be from Becton Dickinson-Pharmingen (San Jose, CA). Frequency and phenotype of T-helper cells (defined as CD3+/CD4+ lymphocytes) and T-cytotoxic cells (CD3+/CD8+ lymphocytes) will be determined by three or four-color flow cytometry. For cell surface staining, 100 μL of anti-coagulated whole blood will be incubated with the corresponding specific fluorescence labelled mAbs for 20 min/RT in the dark. The erythrocytes will be lysed by incubating for 10 min with 2 mL of 1× FACS lysing solution (Becton Dickinson); then, cell suspension will be centrifuged for 5 min at 250 × g, the supernatant will be discarded and the cells will be washed twice with 2 mL of cold PBS at 250 × g/5 min. Finally, cells will be fixed with 250 μL of 2% formaldehyde.

The absolute number of the different T-lymphocyte subpopulations will be calculated on the basis of manually determined total and differential peripheral blood cell counts. For all experiments, appropriate isotype-matched control antibodies will be included. During the acquisition, 5 × 10^4 ^total cells will be analyzed. Dead cells will be gated out by forward and side scatter. Flow cytometry will be performed using the Becton Dickinson FACSORT instrument and analyzed with Cell Quest software.

#### Concentration of serum total cholesterol, transaminases and CPK

The serum concentration of total cholesterol will be measured by colorimetric enzymatic assay; the concentration of hepatic transaminases will be determined by immuno-turbidimetry, while the serum levels of CPK will be determined by a dry chemical assay.

#### Cellular cholesterol measurement

Cellular cholesterol content will be measured with a cholesterol oxidase-based fluorometric assay (Amplex Red Cholesterol Kit) from Molecular Probes (Eugene, OR). Cholesterol content of cells will be normalized to total cellular protein.

Assay to evaluate the adhesive function of LFA-1/ICAM-1: HIV-1 binding to immunoadhesins will be measured in virus capture assays carried out as previously described [23]. High protein-binding 96-well plates (Costar, Cambridge, MA) will be coated with goat anti-human IgG (Fc specific) at 4°C overnight (100 *μ*L/well, 10 *μ*g/mL in 50 mM Tris, pH 9.5). Supernatants will be removed and the plates will be then blocked by adding 200 *μ*L of 3% heat-inactivated bovine serum albumin in PBS to the wells for 1 hr at 37°C. After washing the wells with PBS, 100 *μ*L of supernatants containing ICAM-Ig or VCAM-Ig (6 to 10 mg/mL) will be added to the wells for 1 hr at 37°C. The wells will be washed with cRPMI before adding 100 *μ*L of cell-free virus (20 to 40 ng of p24 per mL) and incubating the plates for 1 hr at 37°C. The wells will be washed three times with cRPMI and 1% Triton X-100 in PBS will be then added to the wells to lyse bound virus. Virus quantitation will be carried out by p24 ELISA on the lysates.

#### Rho activation assay

The Rho activation assay will be performed as described [16]. Briefly, 3 × 10^6 ^peripheral blood mononuclear cells (PBMCs) will be incubated with HIV-1 stocks; then, PBMCs will be washed with ice-cold PBS and lysates will be prepared using Rho activation assay kits (Upstate Biotechnology). GTP-bound Rho will be precipitated with RBD agarose beads, and measured in pellets by Western blot with specific antibodies, using crude cell extracts for normalization.

### Biostatistical considerations

Primary hypothesis: The use of lovastatin (40 mg/day during one year) compared with placebo in HIV-1-infected individuals who are naïve for HAART, should result in a reduction of 50% or higher in viral load and an increase of 20% or higher in CD4 T cell count.

Documentation of outcome and follow-up: Primary and secondary outcome measures will be documented by investigators and research assistants masked to the subject's intervention group assignment.

Sample size and power: Based on a pilot test performed on 40 patients treated at the infectious diseases service, Hospital Universitario San Vicente de Paul (Medellin, Colombia), the baseline values for the primary and secondary outcome measures are as follows:

Mean viral load = 55,000 copies/mL; SD = 80,000 copies/mL

Mean CD4+ T cell count = 500 cells/mL; SD = 180 cells/mL

These outcomes, determined 6 and 12 months after the intervention, are longitudinal data with correlated measurements, and they need special considerations for calculation of power and sample size. In addition to the usual requirements of type I error, type II error, variation measurement and meaningful difference to be detected in longitudinal studies, it is necessary to know the number of repeated observations and an estimate of the correlation among them. The meaningful difference may be expressed as a time-averaged difference in response between groups. Assuming a fixed correlation of 0.8 among the three different measures of viral load, and a time-average difference of 50% between groups (i.e., a decrease of 22,500 copies/mL from the baseline value), with an alpha error of 0.05 and a beta error of 0.2, the sample size required is 55 patients per group. This sample size has more than 90% power to detect an increase of at least 20% in the baseline CD4+ T cell count. There will be not corrections for losses to follow-up or non-adherence, as the study is intended as a pilot phase II trial.

### Interim monitoring

An independent DSMB comprising three members with expertise in statistics, epidemiology and infectious diseases will be responsible for the interim monitoring process. The statistician at the Data-Coordinating Center will be the only person with access to the full database and he will provide the required information, both overall for the interim quality assurance and specific by treatment group (efficacy and safety) exclusively for the DSMB. The first interim monitoring will be conducted when the study participants complete six months of evaluation. The second interim analysis will be conducted when all the patients complete nine months of evaluation. Stopping guidelines for efficacy monitoring will be determined for the primary and secondary outcomes according to the modified O'Brien-Fleming procedure. With this procedure, the values for statistical significance are 0.0006 and 0.0151 in the first (α_1_) and second (α_2_) interim analyses, respectively, for a final significance (α) of 0.0471. Stopping guidelines for safety are left at discretion of the DSMB, with an expected rate of adverse events (hepatic or muscle enzymes elevations) less than 1%. Stopping guidelines regarding futility are not considered and this was agreed in a meeting with the DSMB.

### Analysis plan

The overall efficacy will be established with an intention-to-treat principle, in which the patients will be analyzed in the treatment group to which they were assigned by randomization. The outcomes viral load and CD4 T cells count, as well as most of tertiary/exploratory outcomes, are longitudinal data with repeated measurements and they require special statistical methods to account for the correlation among observations. To analyze this kind of clustered data it is necessary to model both the regression of *Y *(the outcome measure) on *x *(the intervention) and the within-cluster dependence. In a marginal model, the regression of *Y *on *x *and the within-cluster dependence are modelled separately with the application of the statistical method called generalized estimating equations (GEE), which was designed to provide valid regression inferences with correlated data [24].

When the regression analysis for the time-averaged difference in response between groups is the primary interest, as for our research, the *β *coefficient can be estimated by solving the estimating equation



Where *μ*_*i*_(*β*) = *E*(*Y*_*i*_), the marginal expectations for *Y*_*i *_is a vector, which comprises the *m*_*i *_observations (number of measurements) from the *i*^*th *^cluster (each patient). The marginal expectation is the average response over the population of individuals with a common value of *x *(lovastatin or placebo). The covariance matrix, *Cov*(*Y*_*i*_), for *Yi *depends not only on *β *but on α, which characterizes the within-cluster dependence or correlation among observations. This additional problem can be overcome by iterating until convergence between solving  and updating , an estimate of α [24]. This estimate of α may be approximated by the observed correlations in the data and by the assumption of the same correlation among pairs of *m*_*i *_observations (i.e. an exchangeable correlation structure). Additionally, the use of a robust variance estimator (Huber-White) produces valid standard errors even if the correlations within group are not as hypothesized by the specified correlation structure [25]. The GEE approach is simply to choose parameter values  so that the expected *μ*_*i*_(*β*) is as close to the observed *Y*_*i *_as possible, weighting each cluster of data inversely to its variance matrix, *Cov*(*Y*_*i*_; *β*, *α*), which is a function of the within-cluster dependence. These models have been used in the analysis of some interventions to prevent HIV infection in African American adolescents [26], and to evaluate the effect of interventions on outcomes related not only with HIV infection but other sexually transmitted diseases.

The β coefficient obtained with this procedure for the X variable is the estimated time-averaged change in the outcome measure in the lovastatin group compared with placebo, and its p-value is given by the Wald statistic . We will use the *xt *of commands from the statistical package Stata (StataCorp, 2007: Release 10, College Station, Texas, USA) for the proposed analysis of cross-sectional time-series datasets. Specifically, the command *xtgee *will evaluate population-averaged panel-data models using GEE. Although the expectation is that randomization assists in balancing known and unknown prognostic factors in a randomized clinical trial, this assignment process is not a guarantee against the possibility that estimates of treatment effect be modified by covariate imbalance. Accordingly, the estimated time-averaged change in the outcome measure for lovastatin vs. placebo will be adjusted by the most important prognostic factors (baseline CD4+ T cell count, baseline viral load, and adherence to the treatment) considered as additional independent variables in the same GEE model.

### IRB approvals

The protocol and the informed consent document were reviewed and approved by the Ethics Committee responsible for oversight the study (Sede de Investigacion de la Universidad de Antioquia, Medellin, Colombia).

## Discussion

The immunopathogenesis of HIV-1 infection involves multiple interactions between the virus and host's immune system. Although there is *in vivo *activation of both, innate and adaptive immune responses [5], the HIV-1 infection is fought with limited success. In fact, despite of more than two decades of research directed to induce adaptive immune responses to HIV-1, no successful immunological therapy or vaccine has been developed.

The current therapy for HIV-1 infection is HAART, which blocks HIV-1 replication and decreases the incidence of opportunistic infections and mortality [6]. However, the toxicity and long-term side effects of HAART regimens, and the eventual development of resistance have underscored the immediate need for additional therapeutic approaches, which may control effectively the HIV-1 replication and exhibit immunomodulatory properties required to counteract the immune dysregulation observed in chronically HIV-1-infected individuals.

Statins are competitive analogues of the substrate of 3-hydroxy-3-methylglutaryl coenzyme A (HMG-CoA) reductase; this enzyme catalyzes the conversion of hydroxyl-methyl-glutarate into mevalonic acid, a precursor for the biosynthesis of cholesterol and isoprenoids molecules (geranylgeranyl- pyrophosphate and farnesyl- pyrophosphate, two donors of protein prenylation) [27]. Statins are primarily used as hypolipidemic agents for reducing plasma cholesterol levels and, consequently, for preventing cardiovascular diseases; they are considered very safe if not taken in combination with other drugs sharing the same metabolizing pathways. These products are also administered in the treatment of dyslipidemia associated with AIDS and HAART and of lipodystrophy associated with antiretroviral drugs, particularly protease inhibitors (PIs) [9,10]. The beneficial effects of statins also depend on mechanisms other than cholesterol reduction [28]. For example, statins improve endothelial function, reduce blood thrombogenicity, modulate inflammatory responses, and exert recently described immunomodulatory actions [11,28,29].

Four main mechanisms are known to explain the various immunomodulatory effects of statins. The first two are based on the inhibition of HMG-CoA reductase described above. Blockage of the mevalonic pathway reduces synthesis of cholesterol and non-sterol isoprenoid products, leading to cholesterol reduction in plasma and cell membranes (mainly in lipids rafts) [27], and inhibiting prenylation of Rho and Ras GTPases affecting actin cytoskeleton rearrangement and intracellular signalling [16]. The third mechanism is unrelated to inhibition of HMG-CoA reductase, and consist in blocking the interaction between LFA-1 and ICAM-1 adhesion molecules [30], which is crucial to stabilize antigen presenting cell/T-cell contact during antigen presentation and to regulate the traffic of leukocytes during homeostatic and inflammatory conditions [31,32]. Finally, the fourth mechanism is the modulation by statins of surface molecule expression and protein secretion; statins inhibit MHC class-II molecule expression on antigen presenting cells and endothelial cells [13], and CCR5 expression on T lymphocytes [18], while increases the secretion of RANTES and other cytokines [18].

These pleiotropic effects of statins that are related and unrelated to their cholesterol-lowering properties might explain certain positive clinical and laboratory observations, particularly in cardiac and kidney transplantation [33] and ischemic strokes [34]. Few clinical studies have addressed specifically the anti-HIV-1 activity of statins [16,35-38], and some controversial results have been obtained. In the study performed by del Reat et al., six asymptomatic chronically HIV-1-infected patients not receiving HAART were given lovastatin for a month as their only medication [16]. This short-term statin treatment clearly reduced serum viral RNA loads in all patients and increased their CD4 T-cell counts. Discontinuation of treatment was followed by a rebound in viral load.

The study carried-out by Waters et al. evaluated HIV-1-infected individuals on first-line HAART, and showed no statistically significant difference between viral load rebound and blips in patients on stable HAART who did and did not receive statins [38]. They suggested that when patients achieve full viral suppression with their first-line therapy, any small effect of non-HAART medication would be difficult to detect. However, it must be noted that the well known potency of HAART in significantly reducing HIV-1 loads will most likely mask any statin-mediated inhibition of viral production. They concluded that larger studies with longer follow-up and studies in patients with detectable viremia receiving and not receiving HAART are necessary to determine the real viral effect of statins and whether they confer any measurable benefit or harm to patients. On the other hand, other independent study reported that no anti-HIV-1 activity *in vitro *was detected at subtoxic concentrations of several statins (atorvastatin, simvastatin, fluvastatin and lovastatin) [35]. Furthermore, descriptive and not blinded evaluations in HIV-1-infected patients on HAART reported that simvastatin or pravastatin, administered during 12 or 8 weeks respectively, did not induce a significant change in the mean viral load or CD4+ T-cell count [35,36].

The biological effects of statins on cholesterol metabolism, Rho GTPases activity, LFA-1/ICAM-1 function and CCR5/RANTES expression can disrupt several steps of the HIV-1 life cycle, and target cell membrane interaction. HIV-1 binding and entry are achieved through spatial and temporal tuning of protein interactions at the plasma membrane level, which often leads to cell signalling events that favour pathogen infection [39]. Also, numerous studies have shown that HIV-1 requires lipid rafts for several key stages of its replication cycle, and depletion of plasma membrane cholesterol has been shown to inhibit HIV-1 entry and infection in both cell lines and primary cells [14,40]. During HIV-1 infection, CD4 and CCR5 act as the primary cell surface receptor and coreceptor; their engagement by viral gp120 results in T cell activation by recruiting p56lck [41]. It has been shown that CD4 localization in lipid rafts is required for p56lck activation and HIV-1 entry [15]. Cholesterol has also been shown to be critical for CCR5 and CXCR4 conformation and function [42]. On the other hand, HIV-1 binding and internalization into immature dendritic cells have been shown to be dependent on lipid rafts through a cholesterol-dependent pathway [43].

Fusion of viral envelope and plasma membrane of target cells is the step that follows HIV-1 attachment. Even though the mechanism of membrane fusion remains to be fully understood, the cholesterol present in the viral envelope has been shown to play an important role in this process [44]. Removal of cholesterol from mononuclear cells reduces CD4 and CXCR4 co-localization with actin and cell susceptibility to virus-induced membrane fusion [45]. The capacity of HIV-1 to initiate fusion of membranes may rely heavily on the ability of gp41 to bind to cholesterol and get positioned in lipid rafts [46]. The transmembrane envelope gp41 possesses a signal sequence capable of targeting lipid domains thought to be important in gp41-dependent membrane fusion [47].

Cholesterol-dependent events also play a prominent role during later steps in the HIV-1 life cycle, such as assembly, budding and maintenance of virus morphology and infectivity [48]. Cholesterol is an integral constituent of the viral membrane, and thus participates to its structure; the virions being formed must acquire it from the host cell. Cholesterol depletion of HIV-1-infected cells results in a significant decrease of virus release, and the virions released from those cells have little infectious potential [49]. HIV-1 particles can incorporate the adhesion molecule ICAM-1 due to an association between the cytoplasmic domain of ICAM-1 and Pr55Gag; in fact, the physical presence of host-derived ICAM-1 is known to increase virus infectivity [50]. When emerging from their host cell, newly formed viruses must interact again with the plasma membrane of the host cell and lipid rafts have again been shown to be specifically involved in viral budding [51].

The viral regulatory protein Nef has been shown to bind cholesterol, transport newly synthesized cholesterol to the site of viral budding, enrich lipid rafts with newly synthesized cholesterol, increase the formation of lipid rafts and promote the biosynthesis of viruses [52]. Nef expression has been shown to correlate with high viral titers and disease progression; lipid raft-associated Nef has been shown to prime T cell activation through IL-2 secretion resulting from CD3 or CD28 stimulation, promoting HIV-1 replication and virus spread [53].

The molecules LFA-1, ICAM-1, ICAM-2, and ICAM-3 are all expressed on HIV-1-infected cells, and they are also found embedded onto virions [54,55]. The expression levels of these adhesion molecules increases with disease progression [56]. It has been shown that the cell-to cell contact area is largely exploited by the virus. Therefore, the pathogenesis of HIV-1 infection can be modulated by the ICAM-1/LFA-1 interaction through modulatory effects on cell to-cell transmission of HIV-1, virus replication, virus-mediated syncytium formation, depletion of CD4 T cells, and destruction of the architecture of secondary lymphoid organs [57-62].

All the previously cited studies indicate that almost every aspect of the life cycle of HIV-1 relies on cholesterol. In the absence of this steroid, HIV-1 attachment to its host cell is severely impaired since clustering of receptors in lipid rafts is hindered and the conformational state and function of CCR5 and CXCR4 co-receptors are critically affected. In addition virus-cell fusion is greatly diminished, virus transcytosis is inhibited, virus production and budding are reduced, and cell signalling is altered. The effects of cholesterol depletion on the cell cycle are also likely to be most detrimental to HIV-1 biology, bringing virus gene expression to a virtual stop in CD4 T cells. The control of cholesterol through statins is thus likely to interfere with several key steps of HIV-1 replication, offering the possibility of new therapeutic strategies to the current arsenal of antiviral drugs. It would be of high interest to assess the effectiveness of statins to control virus replication based on their ability to affect cholesterol biosynthesis.

Inhibition of the prenylation of Rho GTPases can also regulate the affinity state of LFA-1 and attenuate several aspects of the immune response by modifying the intracellular signalling pathway. Inhibition of prenylation of small GTPases by lovastatin seems to be an alternative mechanism by which this compound might interfere with HIV replication underlying its therapeutic potential during HIV-1 infection [16], as well as for treatment of other inflammatory and immunological disorders [28,29,63,64].

It is known that the hyper-activation status of the immune system plays a pivotal role in the evolution of the disease in HIV-1-infected persons. In order to reduce viral load and immune hyper-activation, statins could also be considered as virostatic agents. At the present time, a very limited number of *in vitro *and *in vivo *studies support the possible anti-HIV-1 activity displayed by statins [16,17,65], but, great amount of data in the literature describe the anti-inflammatory and immunomodulatory effects of statins in various experimental model systems [29,64,66,67].

## Conclusion

Statins could possibly act on distinct levels for controlling HIV-1 replication: a) by lowering cholesterol, they could slow down the cell cycle, modify several signalling pathways and diminish viral entry and budding; b) by inhibiting the prenylation of small GTPases, they could hinder cell movement and decrease expression or affinity level of immune molecules such as LFA-1 and ICAM-1, c) by modulating CCR5 and RANTES expression, they could hamper the infection of target cells by R5 tropic viruses, and d) by blocking LFA-1/ICAMs interaction, they could attenuate several components of inflammatory, immunologic and virological responses. The statins could thus eventually prove to be efficient drugs against HIV-1. These potential antiviral and immunological properties warrant a randomized clinical trial addressed to explore the clinical use of statins in HIV-1 infected patients.

## Abbreviations

AIDS: Acquired Immunodeficiency Syndrome; CPK: Creatine Phosphokinase; DSMB: Data Safety Monitoring Board; GEE: Generalized Estimating Equations; HAART: Highly Active Antiretroviral Therapy; HIV-1: Type-1 Human Immunodeficiency Virus; HMG-CoA: 3-hydroxy-3-methylglutaryl coenzyme A; mAbs: Monoclonal Antibodies; PBMCs: Peripheral Blood Mononuclear Cells.

## Competing interests

Cristina Peñaloza is an employee of Laproff Laboratories, which provided the generic Lovastatin used in this study. The other authors declare that they have no competing interests.

## Authors' contributions

CJM, MTR and FJ conceived the study, participated in its design and drafted the manuscript. EAH, SC, SEM, FG, PA, NG, and CP participated in the design. All authors read and approved the final manuscript.
